# Mobility improvement in the first 6 postoperative weeks in orthogeriatric fracture patients

**DOI:** 10.1007/s00068-021-01856-0

**Published:** 2021-12-21

**Authors:** Alexander M. Keppler, Jenny Holzschuh, Daniel Pfeufer, Johannes Gleich, Carl Neuerburg, Christian Kammerlander, Wolfgang Böcker, Julian Fürmetz

**Affiliations:** 1 Department for Orthopaedics and Trauma Surgery, Muscuoskeletal University Center Munich (MUM), University Hospital LMU Munich, Marchioninistraße 15, 81377 Munich, Germany; 2 AUVA Trauma Hospital Styria, Graz, Austria; 3 Department of Trauma Surgery, BG Trauma Center Murnau, Murnau, Germany

**Keywords:** Proximal femur fracture, Gait speed, Wearabels, Postoperative mobility, Hip fracture rehabilitation, Physical acticity

## Abstract

**Background:**

Physical activity is a relevant outcome parameter in orthopedic surgery, that can be objectively assessed. Until now, there is little information regarding objective gait parameters in the orthogeriatric population. This study focuses on the first 6 weeks of postoperative rehabilitation, and delivers objective data about gait speed and step length in typical orthogeriatric fracture patterns.

**Methods:**

Thirty-one orthogeriatric fracture patients [pertrochanteric femur fractures (PFF), femoral neck (FN), and proximal humerus fractures (PHF)] were consecutively enrolled in a maximum care hospital in a prospective study design. All patients wore an accelerometer placed at the waist during the postoperative stay (24 h/d) and at 6-week follow-up, to measure real gait speed and step length. In addition, self-assessment of mobility (Parker mobility score) and activities of daily living (Barthel index) were collected at baseline, during the inpatient stay, and at 6-week follow-up.

**Results:**

During postoperative hospitalization, significantly higher gait speed (m/s) was observed in the PHF group (0.52 ± 0.27) compared with the FN group (0.36 ± 0.28) and PFF group (0.19 ± 0.28) (*p* < 0.05). Six weeks postoperatively, gait speed improved significantly in all groups (PHF 0.90 ± 0.41; FN 0.72 ± 0.13; PFF 0.60 ± 0.23). Similarly, step length (m) differed between groups postoperatively [FN 0.16 ± 0.13; PFF 0.12 ± 0.15; PHF 0.31 ± 0.05 (*p* < 0.005)] and improved over time significantly (FN 0.47 ± 0.01; 0.39 ± 0.19; 0.50 ± 0.18). Self-assessment scores indicate that the majority of the patients had minor restrictions in mobility before the fracture. These values decreased immediately postoperatively and improved in the first 6 weeks, but did not reach the initial level.

**Conclusions:**

Gait speed, step length, and self-assessment in terms of mobility and activities of daily living improve significantly in the first 6 postoperative weeks in orthogeriatric fracture patients. As very low postoperative mobility during hospitalization was observed, this collective shows great potential in postoperative rehabilitation regardless of their fracture pattern. For this reason, specific aftercare concepts similar to the “fast track” concepts in primary arthroplasty are crucial for orthogeriatric patients in clinical practice.

**Level of evidence:**

Prospective cohort study, 2.

## Introduction

Mobility restoration is one of the main goals of orthopedic surgery and highly relevant in orthogeriatric patients. Suffering a fracture increases level of care in many elderly patients, despite first improvements with interdisciplinary orthogeriatric co-management, which aims to restore the preinjury status [1–3]. One essential part of the treatment is earliest possible and full-weight-bearing mobilization, which reduces mortality especially in proximal femur fracture patients [4–6]. Despite these efforts, overall mortality after hip fracture is still very high and, therefore, orthogeriatric patients need the best postoperative rehabilitation possible [7].

However, previous data on immediate postoperative mobility in orthogeriatric patients often refer to self- or third-party assessment or short walking tests [8, 9]. Continuous monitoring or real-world walking assessment is rare in this patient population, but with modern sensor technologies (wearables), this is easy to implement and provides important information [10, 11].

Accelerometry, a component of modern wearable devices, can be used to calculate various activity parameters through specific algorithms. High-resolution accelerometry (100 Hz) is a simple and robust technique, and can provide parameters like step count or gait speed with the help of suitable algorithms [12]. Gait speed or also walking speed is a promising parameter that is increasingly used to describe overall health status [13–15]. Additionally, reduced gait speed and step length are risk factors for future falls. [16] Thus, accelerometry appears to be a valuable tool for assessing mobility in elderly patients continuously [10, 11, 17].

The aim of the present study was to objectively assess gait parameters using a waist mounted 3D accelerometer during the postoperative in-hospital stay and at the 6 week follow-up. In addition, self-assessment of mobility (Parker Mobility Score) and activities of daily living (Barthel Index) were obtained. Second, the collected data were analyzed to detect fracture related differences. We hypothesized that upper extremity fractures lead to less postoperative immobilization and higher gait speed and step length 6 weeks after trauma.

## Methods

### Study design and participants

Prospective cohort study, Level of Evidence 2.

This study was registered and approved by the Local Ethics Committee (File Number: 17 - 419). Approval was given before the enrollment started. The study followed the Declaration of Helsinki.

**Fig. 1:**
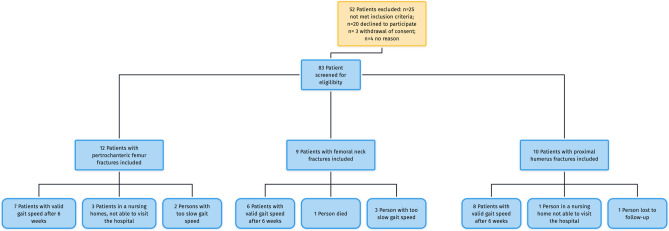
The attrition flowchart depicting application of inclusion and exclusion criteria and withdrawal

Patients meeting the inclusion criteria (age > 65 years, proximal femur fracture or proximal humerus fracture) were consecutively enrolled at a Level I Trauma Center with specialized orthogeriatric care between February 2018 and December 2018. Patients were excluded if the following conditions were present at time of enrollment: immobility prior to surgery (bed ridden patients, severe neurological disorders), dementia, delirium, language barrier, and polytrauma and/or external fixation. 83 patients were screened, and finally, 31 included in the study (Fig. 1). Written consent was obtained from each patient enrolled.

On the first day after surgery, continuous measurement was started for the duration of the hospital stay (24 h/day). Therefore, a waist worn accelerometer (actibelt^®^, Trium Analysis Online GmbH, Munich) was used. The actibelt^®^ has already been used in several clinical studies and is particularly suitable for older patients [10–12].

If, for medical or organizational reasons, an earlier discharge was necessary, the belt was removed earlier. Mobility was assessed with the Parker Mobility Score [18]. Daily activity before fracture was measured with the Barthel Index [19]. Both scores were measured retrospectively on the fifth postoperative day for the baseline level before the fracture, the inpatient stay, and 6 weeks after surgery.

Cognitive impairment, pre- and postfracture mobility, and activities of daily living were assessed with standardized questionnaires. To evaluate cognitive impairment, the Mini-Mental State Examination (MMSE) was performed. Explicit inquiry was used to verify that these questions were understood and answered correctly. A standardized pain regime following the WHO treatment guidelines was used for all patients.

The same physiotherapy staff trained all included patients from the first day after surgery onwards in a regular manner without any additional interventions. Immediate full weight bearing was allowed and no other mobility restrictions were given. All patients received standard postoperative care with possible full weight bearing. If required, patients used different walking aids, according to their needs. Six weeks after surgery, the patients were invited to the clinic for clinical and radiological follow-up. Gait speed and step length were measured with the same 3D accelerometer (actibelt®, Trium Analysis Online GmbH, Munich) under real-world conditions during walking 50 m on a corridor. The BI was also queried again.

### Surgical treatment

Surgical treatment of pertrochanteric fractures was performed by intramedullary nailing [TFNA (Proximal Femoral Nail System); DePuy Synthes, Umkirch, Germany], femoral neck fractures were treated by arthroplasty (total hip replacement: pinnacle acetabular cup, Biolox femoral head and Corail cemented stem; or bipolar hemiarthroplasty, cemented Corail stem; DePuy Synthes, Umkirch, Germany). Among patients with a humeral fracture, patients received either plate or nail osteosynthesis (Philos, Mulitloc nail; DePuy Synthes, Umkirch, Germany) or a reverse shoulder fracture arthroplasty (Wright Medical-Tornier^®^, Burscheid, Germany).

### Statistical analysis

Statistical analysis was performed using SPSS Version 24.0 (IBM AG, Ehningen, Germany), Graphs were created with GraphPad Prism 7 (GraphPad Software, La Jolla, USA). Significances were calculated using an ANOVA or a Mann–Whitney *U* test. A value of *p* < 0.05 was considered significant. Results are expressed as Mean ± SD.

## Results

### Demographics

The mean age differed between the different fracture patterns, but not to a significant level. Comparison of comorbidities based on the Charlson Comorbidity Score showed no significant differences between the three groups (Table 1).

**Table 1:** Patient characteristics and demographic data of the study population

Characteristic	Pertrochanteric femur fracture (*n* = 12)	Femoral neck fracture (*n* = 9)	Proximal humerus fracture (*n* = 10)	*p* Value
BMI (kg/m^2^)	24.2 (± 3.5)	24.0 (± 3.9)	28.8 (± 5.1)	0.025
Age (years)	82.2 (± 6.8)	79.1 (± 7.6)	75.2 (± 6.9)	0.038
Body height (cm)	163.8 (± 5.9)	166.2 (± 5.8)	165.1 (± 6.5)	0.653
Female sex, *n* (%)	11 (91)	9 (100)	9 (90)	
Charlson Comorbidity Score	2.6(± 2.8)	1.7 (± 2.9)	3.2 (± 1.4)	0.43
Length of hospital stay	17 d (± 5.2)	12 d (± 4.5)	9.9 d (± 4.6)	0.01
VAS in rest	2.1 (± 2.2)	0.8 (± 1.3)	0.4 (± 1.1)	0.06
VAS during mobilization	4.3 (± 3.3)	3.0 (± 2.6)	4.6 (± 2.4)	0.39

ANOVA test

*BMI*: body mass index, *VAS*: visual analogue scale for pain

All patients in the PHF group were able to walk free without crutches or walkers, and the patients in the pertrochanteric and femoral neck fracture group were allowed to use crutches or walking frames for the first postoperative days, but full weight bearing was advised if possible.

### Gait speed and step length

**Fig. 2:**
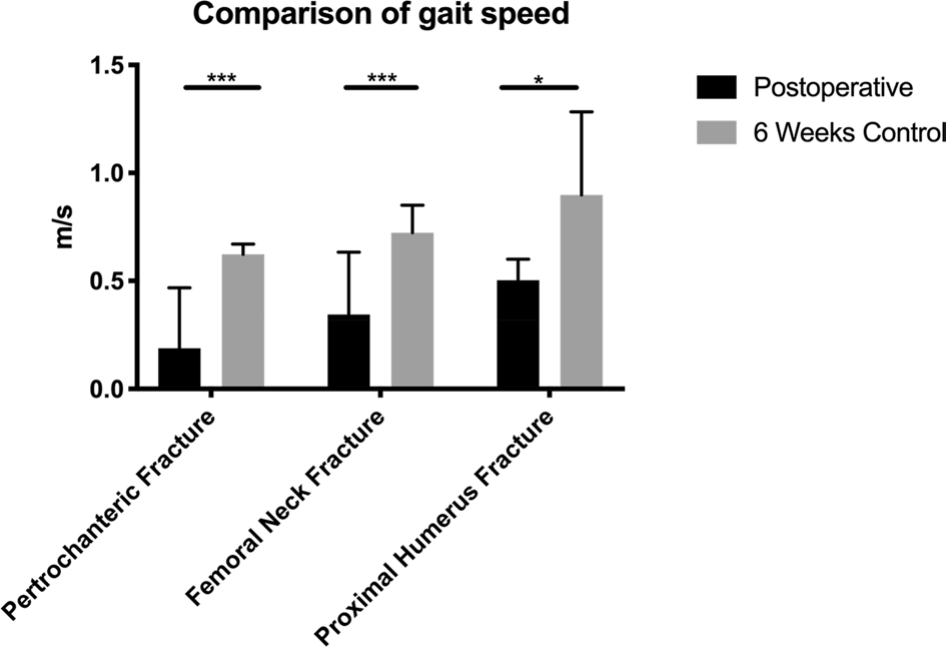
Comparison of gait speed postoperative and 6 weeks after surgery (*U* test; *p < 0.05; **p < 0.01; ***p < 0.001)

The average gait speed of patients (Fig. 2) with a femoral neck fracture during the inpatient stay was 0.36 m/s (± 0.28). In three of nine patients with a femoral neck fracture, no gait speed could be determined, because these patients walked too few steps at a time.

The average gait speed of patients with a pertrochanteric fracture was 0.19 m/s (± 0.28).

Patients with a proximal humerus fracture achieved gait speed of 0.52 m/s (± 0.27) during the in-hospital stay.

Six weeks after trauma, an improvement in gait speed was observed in all patients. Patients with a femoral neck fracture achieved an average gait speed of 0.72 m/s (± 0.13). Patients with a pertrochanteric fracture achieved an average gait speed of 0.60 m/s (± 0.23), while patients with a proximal humerus fracture reached an average of 0.90 m/s ± 0.23).

**Fig. 3:**
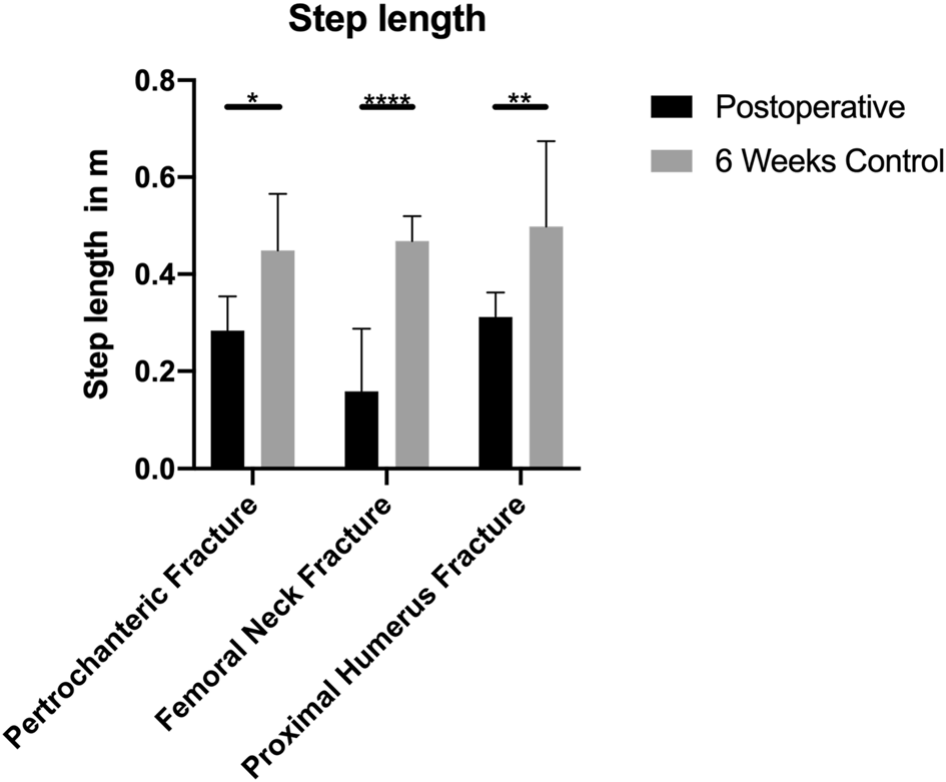
Comparison of gait speed postoperative and 6 weeks after surgery (*U* test; *p < 0.05; **p < 0.01; ***p < 0.001)

The average step length (Fig. 3) in the patients with a femoral fracture was 0.16 m (± 0.13) during the inpatient stay. In three of nine patients, no step length could be determined, because these patients had walked too few steps at a time.

In the group of patients with pertrochanteric fractures, the average step length was 0.28 m (± 0.15). In 2 of 12 patients, no step length could be determined, because these patients walked too few steps at a time.

In comparison, patients with a proximal humerus fracture achieved twice as much step length during the inpatient stay with an average of 0.31 m (± 0.05).

Mean step length improved significantly in all patients after 6 weeks. Femoral neck fracture patients reached 0.47 m (± 0.01), patients with a pertrochanteric fracture 0.45 m (± 0.11). The largest step length was found in patients with a proximal humerus fracture with a value of 0.50 m (± 0.15),

### Geriatric assessment

**Fig. 4:**
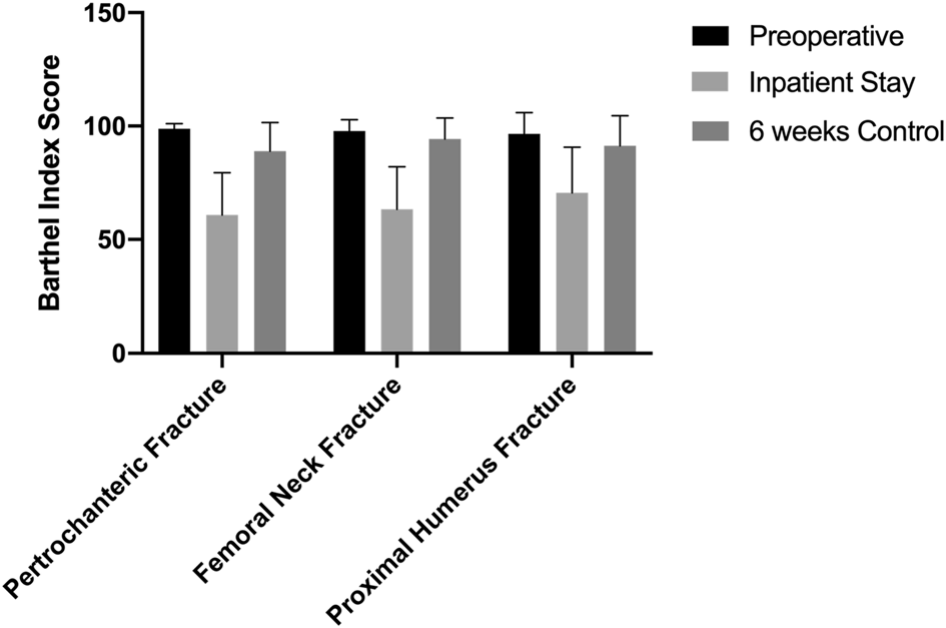
Improvement in geriatric assessment by Barthel Index from inpatient stay to follow-up status after 6 weeks

**Fig. 5:**
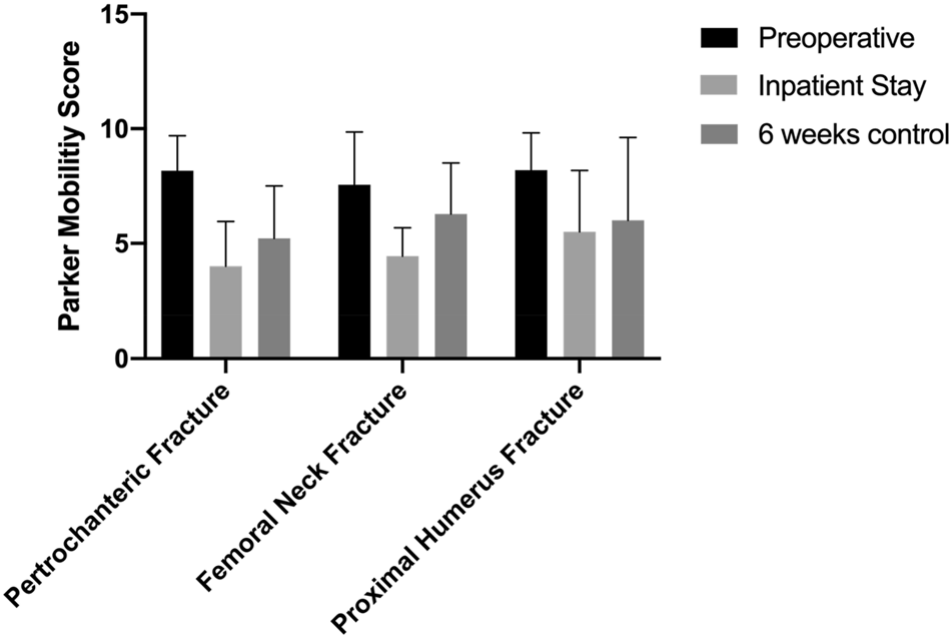
Improvement in Parker Mobility Score from inpatient stay to follow-up status after 6 weeks

Preoperative PMS (range 7.56–8.20) and BI (range 96.5–98.8) indicate that the majority of the patients had minor restrictions in mobility and were not dependent on assistance before the fracture with only minor differences between groups. The differences in BI and PMS improved in the first 6 weeks, but did not reach the initial level (Figs. 4, 5).

## Discussion

This study demonstrates that gait speed and step length improve significantly in the first 6 weeks after surgery in orthogeriatric fracture patients. Self-assessment scores improve similarly, but do not reach prefracture level during this period. Regardless of their fracture pattern, older patients show great potential for recovery during the early postoperative stage. Continuous mobility measurement during the inpatient stay presented very low mobility, impaired gait speed, and step length in all patient groups, despite the possibility and instruction of immediate full weight bearing. This was to be expected for patients with injuries of the lower limbs, but also significant limitations in mobility and gait parameters in patients with a proximal humerus fracture were observed.

There is strong evidence that a delay in surgery of more than 24 h in patients with hip fractures adversely affects outcome in several ways [20, 21]. However, there is little information on how postoperative immobilization negatively affects further physical activity and mortality in orthogeriatric patients, as objective mobility data are missing.

Actions to improve postoperative mobility of orthogeriatric patients are urgently needed. Similar to primary arthroplasty, existing fast track concepts for hip fracture patients showed reduction in length of stay without any rise in readmission or reoperation, but were unable to reduce the high mortality rates of orthogeriatric patients [22–25]. The reported monitoring methods of physical activity are very much characterized by questionnaire-based collection of patient-reported subjective outcome parameters of (PROs and PROMs) [26, 27].

Recent results in a randomized controlled trial indicate that a structured exercise program with 20 additional sessions of physiotherapy at home leads to significant improvement of gait speed in hip fracture patients 4 months postoperatively [28]. There was a high variability of physical activity between days, which indicates that at least 4 consecutive days should be monitored [29]. Therefore, we conclude that objective continuous measurement of mobility during and beyond the inpatient stay in the context of a controlled study is necessary to determine the effect of improved rehabilitation concepts.

The perioperative comprehensive geriatric assessment already shows a reduced risk of mortality and an improvement in physical performance [30, 31]. The recording of peri- and postoperative physical activity in postdischarge care is an indispensable prerequisite for identifying patients at risk and structuring the effective use of existing rehabilitation facilities in an individualized manner.

There are some limitations that have to be considered in this study. For example, the number of participants was very small, which must be considered when looking at the statistical results.

Furthermore, the study population was predominantly female. Although this corresponds to the reality of orthogeriatric care, the majority of orthogeriatric patients are female, this must be taken into account for parameters such as step length.

This study demonstrates that real world and continuous assessment of gait parameters in orthogeriatric patients is possible. Further continuous measurements after the inpatient stay and a longer follow-up are missing in this study. These limitations and a larger sample size will be addressed in ongoing studies.

## Conclusion

Gait speed, step length, and self-assessment in terms of mobility and ADL improve significantly in the first 6 postoperative weeks in orthogeriatric fracture patients. Regardless of their fracture pattern, older patients show great potential for recovery during the early postoperative weeks. For this reason, specific aftercare concepts similar to the “fast track” concepts in primary arthroplasty are crucial for orthogeriatric patients in clinical practice.

## References

[1] Gosch M, Kammerlander C, Roth T, Luger T, Blauth M (1946). Geriatric traumatology: interdisciplinary management of patients with fragility fractures. Dtsch Med Wochenschr.

[2] Kammerlander C, Doshi HK, Böcker W, Gosch M (2016). Fragility fracture care and orthogeriatric comanagement. BioMed Res Int Hindawi.

[3] Killington M, Walker R, Crotty M (2016). The chaotic journey: recovering from hip fracture in a nursing home. Arch Gerontol Geriatr.

[4] Kammerlander C, Pfeufer D, Lisitano LA, Mehaffey S, Böcker W, Neuerburg C (2018). Inability of older adult patients with hip fracture to maintain postoperative weight-bearing restrictions. J Bone Joint Surg.

[5] Schwachmeyer V, Damm P, Bender A, Dymke J, Graichen F, Bergmann G (2013). In vivo hip joint loading during post-operative physiotherapeutic exercises. PLoS ONE.

[6] Siu AL, Penrod JD, Boockvar KS, Koval K, Strauss E, Morrison RS (2006). Early ambulation after hip fracture: effects on function and mortality. Arch Intern Med.

[7] Katsoulis M, Benetou V, Karapetyan T, Feskanich D, Grodstein F, Pettersson-Kymmer U (2017). Excess mortality after hip fracture in elderly persons from Europe and the USA: the CHANCES project. J Int Med.

[8] Kammerlander C, Gosch M, Kammerlander-Knauer U, Luger TJ, Blauth M, Roth T (2011). Long-term functional outcome in geriatric hip fracture patients. Arch Orthop Trauma Surg.

[9] Van Ancum JM, van Schooten KS, Jonkman NH, Huijben B, van Lummel RC, Meskers CGM (2019). Gait speed assessed by a 4-m walk test is not representative of daily-life gait speed in community-dwelling adults. Maturitas.

[10] Mueller A, Hoefling HA, Muaremi A, Praestgaard J, Walsh LC, Bunte O (2019). Continuous digital monitoring of walking speed in frail elderly patients: non-interventional validation study and longitudinal clinical trial. JMIR mHealth uHealth.

[11] Keppler AM, Holzschuh J, Pfeufer D, Neuerburg C, Kammerlander C, Böcker W, et al (2020) Postoperative physical activity in orthogeriatric patients - new insights with continuous monitoring. Injury 10.1016/j.injury.2020.01.041 32033808

[12] Keppler AM, Nuritidinow T, Mueller A, Hoefling H, Schieker M, Clay I (2019). Validity of accelerometry in step detection and gait speed measurement in orthogeriatric patients. Dobkin BH, editor. PLoS ONE.

[13] Studenski S, Perera S, Patel K, Rosano C, Faulkner K, Inzitari M (2011). Gait speed and survival in older adults. JAMA.

[14] Studenski S (2019). Gait speed reveals clues to lifelong health. JAMA Netw Open.

[15] Middleton A, Fritz SL, Lusardi M (2015). Walking speed: the functional vital sign. J Aging Phys Act.

[16] Verghese J, Holtzer R, Lipton RB, Wang C (2009). Quantitative gait markers and incident fall risk in older adults. J Gerontol A Biol Sci Med Sci.

[17] Rojer AGM, Coni A, Mellone S, Van Ancum JM, Vereijken B, Helbostad JL (2021). Robustness of in-laboratory and daily-life gait speed measures over one year in high functioning 61- to 70-year-old adults. Gerontology.

[18] Parker MJ, Palmer CR (1993). A new mobility score for predicting mortality after hip fracture. J Bone Jt Surg Br Vol.

[19] Mahoney FI, Barthel DW (1965). Functional evaluation: the Barthel Index. Md State Med J.

[20] Hongisto MT, Nuotio MS, Luukkaala T, Väistö O, Pihlajamäki HK (2019). Delay to surgery of less than 12 hours is associated with improved short- and long-term survival in moderate- to high-risk hip fracture patients. Geriatr Orthop Surg Rehab.

[21] Pincus D, Ravi B, Wasserstein D, Huang A, Paterson JM, Nathens AB (2017). Association between wait time and 30-day mortality in adults undergoing hip fracture surgery. JAMA.

[22] Pollmann CT, Røtterud JH, Gjertsen J-E, Dahl FA, Lenvik O, Årøen A (2019). Fast track hip fracture care and mortality—an observational study of 2230 patients. BMC musculoskeletal disorders. BioMed Central.

[23] Gomez M, Rony L, Marc C, Talha A, Ruiz N, Noublanche S (2020). Fast-track care for pertrochanteric hip fracture: what impact on function and autonomy after discharge?. Orthop Traumatol Surg Res.

[24] Gomez M, Marc C, Talha A, Ruiz N, Noublanche S, Gillibert A (2019). G Model Fast track care for pertrochanteric hip fractures: how does it impact length of stay and complications?. Orthop Traumatol Surg Res.

[25] Gromov K, Willendrup F, Palm H, Troelsen A, Husted H (2015). Fast-track pathway for reduction of dislocated hip arthroplasty reduces surgical delay and length of stay. Acta Orthop.

[26] Grant S, Blom AW, Craddock I, Whitehouse M, Gooberman-Hill R (2019). Home health monitoring around the time of surgery: qualitative study of patients’ experiences before and after joint replacement. BMJ Open.

[27] Hoogland J, Wijnen A, Munsterman T, Gerritsma CLE, Dijkstra B, Zijlstra WP (2019). Feasibility and patient experience of a home-based rehabilitation program driven by a tablet app and mobility monitoring for patients after a total hip arthroplasty. JMIR mHealth uHealth.

[28] Taraldsen K, Thingstad P, Døhl Ø, Follestad T, Helbostad JL, Lamb SE (2019). Short and long-term clinical effectiveness and cost-effectiveness of a late-phase community-based balance and gait exercise program following hip fracture. The EVA-Hip Randomised Controlled Trial. PLoS ONE.

[29] Taraldsen K, Vereijken B, Thingstad P, Sletvold O, Helbostad JL (2014). Multiple days of monitoring are needed to obtain a reliable estimate of physical activity in hip-fracture patients. J Aging Phys Act.

[30] Prestmo A, Hagen G, Sletvold O, Helbostad JL, Thingstad P, Taraldsen K (2015). Comprehensive geriatric care for patients with hip fractures: a prospective, randomised, controlled trial. Lancet.

[31] Eamer G, Taheri A, Chen SS, Daviduck Q, Chambers T, Shi X (2018). Comprehensive geriatric assessment for older people admitted to a surgical service. Cochrane Database Syst Rev.

